# Anti-Oppressive Practice and Reflexive Lifeworld-Led Approaches to Care: A Framework for Teaching Nurses about Social Justice

**DOI:** 10.1155/2015/187508

**Published:** 2015-03-08

**Authors:** Jacqueline Sarah Hutchison

**Affiliations:** Faculty of Health and Social Care, Hull University, Hull HU6 7XR, UK

## Abstract

This paper was initially written for a European Academy of Caring Science workshop and aimed to provide clarity and direction about Caring Science by offering some ideas emerging from the philosophy, themes, and projects of EACS. An underpinning concept for the work of the Academy is the lifeworld. The focus of the workshop was to explore the lifeworld of the patient, student, and carer. The intention was to promote discussion around the need to provide alternative ways to conceptualise caring relevant knowledge, naming phenomena and practices central to caring sciences, and the educational curriculum and its adequacy for caring science. This paper seeks to identify concepts and approaches to understanding oppression, power, and justice which enable nurses to challenge the structures in health care environments which discriminate or disempower clients. Anti-oppressive practice theory and reflexive lifeworld-led approaches to care enable nurses to be critical of their practice. A framework for teaching social justice in health care is offered to augment teaching students to challenge oppressive practice and to assist nurses to reflect and develop conceptual models to guide practices which are central to promoting caring interactions.

## 1. Introduction

Teaching students about phenomena which enable anti-oppressive practice (AOP) presents both a challenge and an opportunity to help students see beyond everyday events and consider how their own practice and the environments in which they work may disable patients and even discriminate against them. The concept of AOP developed from the new social movements in the 1960's which focused on human rights and social justice. Debates in social work advocated the use of AOP theory [1–6] and have established an emerging agenda for AOP or anti-discriminatory practice in health care. Discussions around social justice can be used to engage the nurse in a reflexive debate around their own practice, the practices of others, and the social, economic, and cultural milieu in which care takes place. The aim of this paper is to outline and consider how an AOP model and a reflexive lifeworld-led approach to care, can be used as a framework to augment teaching which focuses on educating students to practice in ways, that challenge oppressive structures or behaviours in health care.

## 2. Background: Agenda for AOP

I argue that debates that consider structures of power and oppression are timely. Recent political and social events, as well as events in health care, jeopardise the capacity for nurses to treat patients fairly and equitably. Within Europe, we have seen contracting economic growth, increased unemployment, and job losses and Anti-European Union parties have gained ground in so many countries. The former European Commission President, Jose Manuel Barroso, warned against nationalism, xenophobia, and racism ahead of European Parliament elections last year [7]. Numerous national protest parties have emerged across Europe. In the European elections right-wing parties with strong positions on immigration did well in several countries, including United Kingdom (UK), France, Italy, and Greece. In Greece and Italy, far-left movements have strong popular backing, as well as single-issue parties such as Germany's anti-euro, Alternative für Deutschland party (AfD), who secured representation in the 760-seat European Parliament, (with 7% of the vote) the European Union's (EU) only directly elected body.

We have seen political and social movements in Europe galvanised in the wake of the economic crisis and threats of terror. Nursing's renewed interest in social justice has come at a time when disparities between rich and poor have widened. The Organization for Economic Cooperation and Development (OECD) published figures that show that within its 34 member countries, which include countries from North and South America to Europe and the Asia-Pacific region, the average income of the richest 10% of the population is about 9.5 times that of the poorest 10%. This is the highest level of disparity for thirty years [8].

In health care, reports of patient neglect have become a public concern throughout Europe and in the US [9]. In the UK a number of high profile scandals in health care have highlighted the importance of tacking oppression and the misuse of power in health care settings. Many of these scandals involve a degree of patient neglect with patients being left malnourished, dehydrated, in pain, or unwashed. The recent publication of the Francis Report in the UK in 2013 [10], provoked a national debate about nursing and its inherent professional values. Between January 2005 and March 2009, approximately 1200 patients were reported to have died as a direct result of the poor care they received at the Mid Staffordshire NHS Foundation Trust. The public enquiry that followed highlighted failures to care delivery. These events were seen, in part, to be the consequence of poor leadership, a tolerance of poor standards, and a culture that prized meeting targets, many of which were financial, rather than delivering good quality care. Staff were fearful of repercussions for themselves, if situations that resulted in poor care were challenged.

Given this current climate, there has rarely been a better time to ensure we are teaching values to nurses which promote justice, equality, and fairness. Debates which draw attention to practices and structures of oppression are essential and should be central to education and practice in nursing if we are to preserve core values which underpin caring relevant knowledge. It is imperative that nurses are taught concepts which are applicable to contemporary caring contexts and which can respond to changing global political contexts.

## 3. Social Justice in Nursing

The concept of social justice is not clearly defined in the literature [11] but it is commonly considered to involve the relationship between society and the individual and a balance between the benefits and burdens for all citizens, resulting in fairness and equity [12]. In striving for social justice, we are concerned with what the individual owes to the community and vice versa. The nature of social justice focuses on the collective interests of members of communities, rather than the individual concerns of one person for another. A concept analysis by Buettner-Schmidt and Lobo highlights the paucity of nursing literature around justice and that nurses' concern with social justice has diminished until recently, as debates about providing individualised care became more significant in the literature [11].

Whilst there are numerous dimensions to social justice which concern policy makers, nurses, doctors, social workers, and politicians differently, justice in health care principally involves the problem of distributive justice (the sharing of materials, resources, and services) and the idea of procedural justice (maintaining fairness when making decisions). As health care systems, in the main, operate in conditions of scarcity, the allocation of resources in health care is of considerable public concern [12].

Nursing theories and models have been less radical than social work models, in challenging structures of oppression [11]. Traditional nursing models have focused on biological models, holistic care, individualised care, self-care, and patient centred care [13–16]. These models often consider the relationship between patient and nurse and developmental, environmental, social, cultural, and psychological contexts for care. More recent nursing models do incorporate social justice as a concept for analysis. Fawcett and Russell [17] stress the need for nurses to participate in the field of policy and offer a conceptual model for nursing practice which considers social justice as a core nursing value and part of an underpinning philosophy which aims to promote an interactive process that engages nurses in the development and implementation of health policy. Other nurse theorists discuss social justice as part of their conceptual models as a prerequisite to sustain compassionate and caring practices and a challenge to the social inequalities which cause suffering for individuals and communities [18–20].

**Figure 1:**
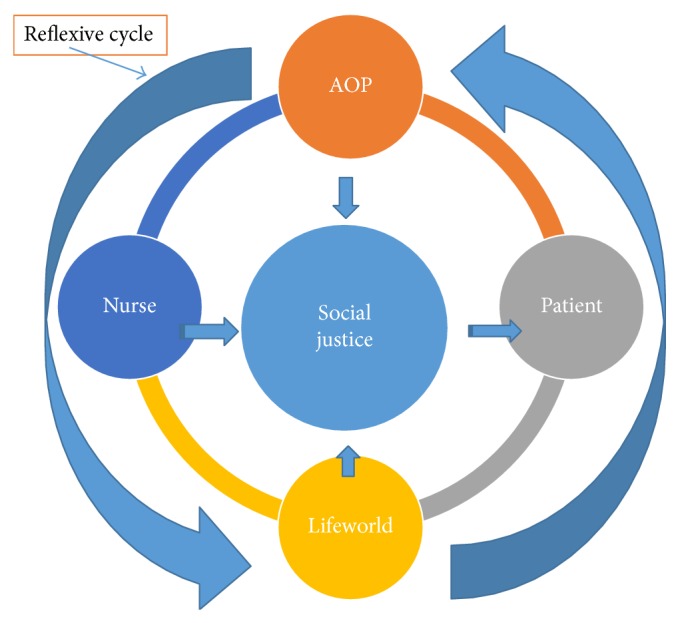
Social justice model.

These models, however, do not assist nurses to understand the distribution of power in communities, allocation of resources, the institutions, systems, processes, and polices which influence the nature of health care. Whilst some models of nursing have incorporated elements of social justice, a clear framework which focuses on social justice does not exist and a much more comprehensive approach to tackling oppression is needed. A conceptual framework, which focuses on both the practices of nurses and the structures within which nurses operate, has the potential to offer a far more comprehensive approach to tackling oppression (see Figure 1).

## 4. AOP Models

Through an analysis of power and oppression, AOP theory offers a radical approach to challenge structural inequalities and practices of oppression. Informed by social work theory, it seeks to address the structural inequalities and divisions experienced by clients. AOP philosophy emphasizes equality of outcome and empowerment of individuals by utilising current legislation in an informed and knowledgeable way. Practices that marginalized clients can be identified and oppressive practices can be transformed. The client's knowledge is recognised as a source of expertise [21] and promoting clients' agency, in order that they may exercise control over decision-making processes in relation to their care, is considered. AOP theory attempts to place a spotlight, not only on the lives of clients and our own lives, but on the historical and sociopolitical contexts in which we live.

AOP, however, can be criticised for being a theory that focuses primarily on structural understandings of power and on “power over” strategies [22]. Whilst acknowledging and challenging structural dimensions to oppression are essential, this approach can appear deterministic and portray the client as victim. Wilson and Beresford suggest that, despite aspiring to empower clients, AOP models have failed to involve service users themselves in the development of anti-oppressive theory and practice, furthermore, they suggest there is little evidence that proponents of AOP have considered their own role politically as being part of the structures in social care that may discriminate or oppress clients [23]. A reflexive lifeworld-led approach to social justice offers a solution to these apparent contradictions.

## 5. The Lifeworld

In contrast to tackling structures of oppression in society, nursing practice needs also to be cognisant of critiques of dominant medical discourses informed by listening and learning from clients. By listening to clients stories we learn how they experience their bodies and how they make sense of their suffering or their wellness. The concept of the lifeworld is concerned with the subjective nature of being. It has its origins in the work of Husserl and the “lebenswelt” or “lifeworld” which is the phenomena of enquiry in studies which focus on the often unquestioned experiences of individuals [24]. Husserl's work was developed further by both Merleau Ponty, [25] which gave emphasis to the body as the primary sight of knowing, and by Heidegger's philosophy which focused on the study of being [26]. Lived experience can offer direct insights for caring science and education for caring science.

The notion of lifeworld-led care that is developed by Dahlberg et al. [27] involves three dimensions: a philosophy of the person, a view of wellbeing, and a philosophy of care that is focused on the individual's experience. Lifeworld-led approaches to care recognise the importance of promoting humanising philosophies in care in a world of increasing technological progress. Todres et al. advocate lifeworld-led approaches to care as antidote to dehumanising forces inherent in technological progress [28]. Drawing from Husserl's existential phenomenological tradition, the need to put human experiences at the centre of any caring framework is explored. A political focus is also stressed if care is to be informed at both practice and policy levels.

The challenge of teaching nursing students about practices that are oppressive lies in the every-day-ness of the working environment that we inhabit. Behaviours, attitudes, and beliefs that are common in health environments can obscure the nature of events and the need to critique these common experiences and be mindful of how practitioners can perpetuate structures of oppressive. Teaching students about social justice involves helping students to see beyond the everyday and consider how their own practice, and the environments in which they work, may disable clients and even discriminate against them. Husserl considered the Lifeworld as fundamental for all epistemological enquiry and the nurse's role puts them in key positions to listen to and appreciate patient's experiences.

Lifeworld-led approaches offer a solution to this problem of everydayness by unveiling day-to-day experiences which marginalise and isolate clients. Listening to client's experiences and acknowledging their pain and suffering can provide a more tangible platform from which we consider client's needs. Furthermore lifeworld-led approaches emphasise the power clients have to identify their own needs and transform their own lives. The power in collective action through sharing experiences, listening to clients, and enabling them to take decisions is prioritised. The advantage of teaching lifeworld approaches alongside AOP theory is that this enables students to appreciate the need to critique the day-to-day events both they and their patients' experience and the taken-for-granted nature of the health care environments they inhabit. I argue that AOP and lifeworld-led approaches to care offer a more comprehensive framework to tackling oppression from both personal and political dimensions.

## 6. Reflexivity and Developing a Framework for Social Justice

Reflexivity involves recognising the inherent bias the researcher brings to their research and that it is impossible to isolate the affects the researcher will have on the research process. The reflexive process in social research involves understanding and reflecting on that process [29]. Debates in anthropology around reflexivity have emerged as a result of the anthropologist's position in the post-colonial period and a growing appreciation that anthropologists had failed to consider the effects of colonialism both on the people they had studied and on the research process itself [30–32]. The distinction between researcher and the researched, highlighted in anthropological theory, can be exploited to better understand relationships between the nurse and patient. The nurse too has to recognise and acknowledge the context of their interactions with patients, their own political and sociocultural circumstances, the theoretical frameworks within which they work, and the relationships they develop and the affects these variables have on the patient's experience.

Our theories of the world around us, are informed by our experiences and interactions with others, for example, our experiences of being; white, female and heterosexual. A reflexive approach helps us respond to the reality of our own experiences in the world and the impact this may have on those we provide care for. The lifeworld of patients and health care professionals can be quite different, and understanding this requires a reflexivity of self and others in the world and a consideration of the cultural, political, social, and economic conditions of people's lives and the environments in which health care takes place. Encouraging reflectivity, which moves beyond the everyday and taken-for-granted assumptions made about our lives and others and takes into account the complexity of the milieu in which nurses operate, helps us to begin to see the barriers to providing care which is sensitive and anti-oppressive (see Figure 1).

## 7. Methods for Teaching Students AOP

Methods for teaching student nurses about AOP can be simple but need to be multidimensional and take into account the institutions, structures, processes, and policies which shape health care environments as well as the patient experience and the experience of the nurse. An understanding of how health policy is formulated and reflects the choices and decisions made by governments about resource allocation is essential. Nurse's critiques of practice need to engage with the political contexts which shape caring interactions. To understand dimensions that influence power relations in health, Student's also need to have a fundamental grasp of the key sociological concepts such as class, race, sexuality, gender, age, and disability which influence the form and nature of social institutions.

A number of authors have suggested that nurses have historically been focused on understanding and caring for individuals rather than communities and social groups; therefore, nursing curricula should focus on broadening student political participation [33, 34]. However, how these more abstract concepts are social constructed and embedded in our day-to-day experiences can be a difficult concept to demonstrate. It is difficult to understand how health services, cultures, and processes may fail to meet the needs of patients because sometimes the effects of these inequalities may not be evident.

Simple vignettes that illustrate a variety of everyday patient/s experiences of care can form the basis for a reflective exercise which enables students to see the simple interactions between nurses and patients that can be oppressive. Seemingly simple nurse, patient interactions such as not listening to patient's requests, talking over patients, and not prioritising the patient's privacy or their need to make decisions for themselves, constitutes oppressive practice. The student can see directly the impact their behaviour and attitudes can have on the patient experience. As an initial exercise to help students grasp the basic concepts of oppressive practice, this method has considerable resonance with nurses as it is often the day-today interaction with patients that nurses are concerned with. A reflexive and multidimensional approach to social justice enables the nurse to have a critical lifeworld-led perspective in order that they can challenge oppressive structures and practices which affect patients and carers.

## 8. Conclusion

It is imperative that knowledge that is relevant for caring in contemporary contexts is developed to support nursing curricula. Nurse curricula need to respond to changing contemporary global political and social climates. Many of the current concepts around social justice which are used to support nurse curricula focus on equality and diversity and do not go far enough to reveal dimensions of power and challenge structures of oppression.

An educational curriculum that is adequate for caring science needs to offer frameworks for practice which are robust and enable practitioners to challenge structures of oppression. By utilising lifeworld-led philosophies and an AOP model to promote a reflexivity of self, a framework to guide practice can be developed. Concepts that focus both on patient experiences and everyday praxis, as well as political and social structures, offer a more relevant way of conceptualising oppression. The framework can unveil practices sometimes hidden in everyday-ness, to reveal structures in health care that oppress and discriminate against those we provide care for. There is a need to consider both top-down and bottom-up models of oppression so that we can learn from the personal and political contexts of people's lives. Adopting a framework utilising AOP theory and reflexive lifeworld-led approaches to care, ensures nurses continually challenge how values, social difference, and power affect our interaction with others and our experience of the world.
